# A Genome-Wide Screen for Interactions Reveals a New Locus on 4p15 Modifying the Effect of Waist-to-Hip Ratio on Total Cholesterol

**DOI:** 10.1371/journal.pgen.1002333

**Published:** 2011-10-20

**Authors:** Ida Surakka, Aaron Isaacs, Lennart C. Karssen, Pirkka-Pekka P. Laurila, Rita P. S. Middelberg, Emmi Tikkanen, Janina S. Ried, Claudia Lamina, Massimo Mangino, Wilmar Igl, Jouke-Jan Hottenga, Vasiliki Lagou, Pim van der Harst, Irene Mateo Leach, Tõnu Esko, Zoltán Kutalik, Nicholas W. Wainwright, Maksim V. Struchalin, Antti-Pekka Sarin, Antti J. Kangas, Jorma S. Viikari, Markus Perola, Taina Rantanen, Ann-Kristin Petersen, Pasi Soininen, Åsa Johansson, Nicole Soranzo, Andrew C. Heath, Theodore Papamarkou, Inga Prokopenko, Anke Tönjes, Florian Kronenberg, Angela Döring, Fernando Rivadeneira, Grant W. Montgomery, John B. Whitfield, Mika Kähönen, Terho Lehtimäki, Nelson B. Freimer, Gonneke Willemsen, Eco J. C. de Geus, Aarno Palotie, Manj S. Sandhu, Dawn M. Waterworth, Andres Metspalu, Michael Stumvoll, André G. Uitterlinden, Antti Jula, Gerjan Navis, Cisca Wijmenga, Bruce H. R. Wolffenbuttel, Marja-Riitta Taskinen, Mika Ala-Korpela, Jaakko Kaprio, Kirsten O. Kyvik, Dorret I. Boomsma, Nancy L. Pedersen, Ulf Gyllensten, James F. Wilson, Igor Rudan, Harry Campbell, Peter P. Pramstaller, Tim D. Spector, Jacqueline C. M. Witteman, Johan G. Eriksson, Veikko Salomaa, Ben A. Oostra, Olli T. Raitakari, H.-Erich Wichmann, Christian Gieger, Marjo-Riitta Järvelin, Nicholas G. Martin, Albert Hofman, Mark I. McCarthy, Leena Peltonen, Cornelia M. van Duijn, Yurii S. Aulchenko, Samuli Ripatti, for the ENGAGE Consortium

**Affiliations:** 1 Institute for Molecular Medicine Finland (FIMM), University of Helsinki, Helsinki, Finland; 2 Public Health Genomics Unit, National Institute for Health and Welfare, Helsinki, Finland; 3 Genetic Epidemiology Unit, Department of Epidemiology, Erasmus University Medical Center, Rotterdam, The Netherlands; 4 Centre for Medical Systems Biology, Netherlands Genomics Initiative, Leiden, The Netherlands; 5 Department of Medical Genetics, Haartman Institute, University of Helsinki and Helsinki University Central Hospital, Helsinki, Finland; 6 Queensland Institute of Medical Research, Brisbane, Australia; 7 Department of Medicine, Prince Charles Hospital, Chermside, Australia; 8 Institute of Genetic Epidemiology, Helmholtz Zentrum München – German Research Center for Environmental Health, Neuherberg, Germany; 9 Division of Genetic Epidemiology, Department of Medical Genetics, Molecular and Clinical Pharmacology, Innsbruck Medical University, Innsbruck, Austria; 10 Department of Twin Research and Genetic Epidemiology, King's College London, London, United Kingdom; 11 Department of Immunology, Genetics, and Pathology, University of Uppsala, Uppsala, Sweden; 12 Department of Biological Psychology, VU University Amsterdam, Amsterdam, The Netherlands; 13 Oxford Centre for Diabetes, Endocrinology, and Metabolism, University of Oxford, Oxford, United Kingdom; 14 Wellcome Trust Centre for Human Genetics, University of Oxford, Oxford, United Kingdom; 15 Department of Cardiology, University Medical Center Groningen, University of Groningen, Groningen, The Netherlands; 16 The Estonian Genome Center and the Center of Translational Genomics of the University of Tartu, Tartu, Estonia; 17 The Institute of Molecular and Cellular Biology of the University of Tartu, Tartu, Estonia; 18 Department of Medical Genetics, University of Lausanne, Lausanne, Switzerland; 19 Swiss Institute of Bioinformatics, Lausanne, Switzerland; 20 Genetic Epidemiology Group, Wellcome Trust Sanger Institute, Hinxton, United Kingdom; 21 Non-Communicable Disease Research Group, Department of Public Health and Primary Care, University of Cambridge, Cambridge, United Kingdom; 22 Computational Medicine Research Group, Institute of Clinical Medicine, University of Oulu and Biocenter Oulu, Oulu, Finland; 23 Department of Medicine, University of Turku and Turku University Hospital, Turku, Finland; 24 Department of Health Sciences, Gerontology Research Centre, University of Jyväskylä, Jyväskylä, Finland; 25 NMR Metabonomics Laboratory, Department of Biosciences, University of Eastern Finland, Kuopio, Finland; 26 Department of Psychiatry, Washington University School of Medicine, St. Louis, Missouri, United States of America; 27 Medical Department, University of Leipzig, Leipzig, Germany; 28 IFB AdiposityDiseases, University of Leipzig, Leipzig, Germany; 29 Institute of Epidemiology I, Helmholtz Zentrum München – German Research Center for Environmental Health, Neuherberg, Germany; 30 Institute of Epidemiology II, Helmholtz Zentrum München – German Research Center for Environmental Health, Neuherberg, Germany; 31 Department of Epidemiology, Erasmus MC, Rotterdam, The Netherlands; 32 Department of Internal Medicine, Erasmus MC, Rotterdam, The Netherlands; 33 Netherlands Genomics Initiative (NGI)–sponsored Netherlands Consortium for Healthy Aging (NCHA), Leiden, The Netherlands; 34 Department of Clinical Physiology, Tampere University Hospital, Tampere, Finland; 35 Medical School, University of Tampere, Tampere, Finland; 36 Department of Clinical Chemistry, Tampere University Hospital, Tampere, Finland; 37 Department of Psychiatry, University of California Los Angeles, Los Angeles, United States of America; 38 Center for Neurobehavioral Genetics, Semel Institute for Neuroscience and Human Behavior, University of California Los Angeles, Los Angeles, United States of America; 39 The Broad Institute of Massachusetts Institute of Technology and Harvard University, Cambridge, Massachusetts, United States of America; 40 Genetics, Medicines Discovery, and Development, GlaxoSmithKline, Philadelphia, Pennsylvania, United States of America; 41 The Estonian Biocentre, Tartu, Estonia; 42 Department of Medicine, University of Leipzig, Leipzig, Germany; 43 Department of Chronic Disease Prevention, National Institute for Health and Welfare, Turku, Finland; 44 Division of Nephrology, Department of Internal Medicine, University Medical Center Groningen, University of Groningen, Groningen, The Netherlands; 45 Department of Genetics, University Medical Centre Groningen and University of Groningen, Groningen, The Netherlands; 46 Department of Endocrinology, University Medical Center Groningen, University of Groningen, Groningen, The Netherlands; 47 Department of Medicine, Helsinki University Central Hospital, Helsinki, Finland; 48 Department of Internal Medicine and Biocenter Oulu, Clinical Research Center, University of Oulu, Oulu, Finland; 49 Department of Public Health, University of Helsinki, Helsinki, Finland; 50 Unit for Child and Adolescent Mental Health, National Institute for Health and Welfare, Helsinki, Finland; 51 Institute of Regional Health Services Research, University of Southern Denmark, Odense, Denmark; 52 Odense Patient data Explorative Network (OPEN), Odense University Hospital, Odense, Denmark; 53 Department of Medical Epidemiology and Biostatistics, Karolinska Institutet, Stockholm, Sweden; 54 Centre for Population Health Sciences, University of Edinburgh Medical School, Edinburgh, United Kingdom; 55 Croatian Centre for Global Health, University of Split Medical School, Split, Croatia; 56 Institute of Genetic Medicine, European Academy Bozen/Bolzano (EURAC), Bolzano, Italy; 57 Affiliated Institute of the University of Lübeck, Lübeck, Germany; 58 Department of Neurology, University of Lübeck, Lübeck, Germany; 59 Department of Neurology, Central Hospital of Bolzano, Bolzano, Italy; 60 Department of General Practice and Primary Health Care, University of Helsinki, Helsinki, Finland; 61 Unit of General Practice, Helsinki University Central Hospital, Helsinki, Finland; 62 Folkhälsan Research Centre, Helsinki, Finland; 63 Vasa Central Hospital, Vasa, Finland; 64 Department of Health Promotion and Chronic Disease Prevention, National Institute for Health and Welfare, Helsinki, Finland; 65 Unit of Chronic Disease Epidemiology and Prevention, National Institute for Health and Welfare, Helsinki, Finland; 66 Department of Clinical Genetics, Erasmus MC, Rotterdam, The Netherlands; 67 Research Centre of Applied and Preventive Cardiovascular Medicine, University of Turku, Turku, Finland; 68 Clinical Physiology, University of Turku and Turku University Hospital, Turku, Finland; 69 Institute of Medical Informatics, Biometry and Epidemiology, Chair of Epidemiology, Ludwig-Maximilians-Universität, Munich, Germany; 70 Klinikum Grosshadern, Munich, Germany; 71 Department of Epidemiology and Biostatistics, School of Public Health, Imperial College London, London, United Kingdom; 72 Oxford NIHR Biomedical Research Centre, Churchill Hospital, Oxford, United Kingdom; Georgia Institute of Technology, United States of America

## Abstract

Recent genome-wide association (GWA) studies described 95 loci controlling serum lipid levels. These common variants explain ∼25% of the heritability of the phenotypes. To date, no unbiased screen for gene–environment interactions for circulating lipids has been reported. We screened for variants that modify the relationship between known epidemiological risk factors and circulating lipid levels in a meta-analysis of genome-wide association (GWA) data from 18 population-based cohorts with European ancestry (maximum *N* = 32,225). We collected 8 further cohorts (*N* = 17,102) for replication, and *rs6448771* on 4p15 demonstrated genome-wide significant interaction with waist-to-hip-ratio (WHR) on total cholesterol (TC) with a combined *P*-value of 4.79×10^−9^. There were two potential candidate genes in the region, *PCDH7* and *CCKAR*, with differential expression levels for *rs6448771* genotypes in adipose tissue. The effect of WHR on TC was strongest for individuals carrying two copies of G allele, for whom a one standard deviation (sd) difference in WHR corresponds to 0.19 sd difference in TC concentration, while for A allele homozygous the difference was 0.12 sd. Our findings may open up possibilities for targeted intervention strategies for people characterized by specific genomic profiles. However, more refined measures of both body-fat distribution and metabolic measures are needed to understand how their joint dynamics are modified by the newly found locus.

## Author Summary

Circulating serum lipids contribute greatly to the global health by affecting the risk for cardiovascular diseases. Serum lipid levels are partly inherited, and already 95 loci affecting high- and low-density lipoprotein cholesterol, total cholesterol, and triglycerides have been found. Serum lipids are also known to be affected by multiple epidemiological risk factors like body composition, lifestyle, and sex. It has been hypothesized that there are loci modifying the effects between risk factors and serum lipids, but to date only candidate gene studies for interactions have been reported. We conducted a genome-wide screen with meta-analysis approach to identify loci having interactions with epidemiological risk factors on serum lipids with over 30,000 population-based samples. When combining results from our initial datasets and 8 additional replication cohorts (maximum *N* = 17,102), we found a genome-wide significant locus in chromosome 4p15 with a joint *P*-value of 4.79×10^−9^ modifying the effect of waist-to-hip ratio on total cholesterol. In the area surrounding this genetic variant, there were two genes having association between the genotypes and the gene expression in adipose tissue, and we also found enrichment of association in genes belonging to lipid metabolism related functions.

## Introduction

Serum lipids are important determinants of cardiovascular disease and related morbidity [1]. The heritability of circulating lipid levels is estimated to be 40%–60% and recent genome-wide association (GWA) studies implicated a total of 95 loci associated with serum high-density lipoprotein cholesterol (HDL-C), low-density lipoprotein cholesterol (LDL-C), total cholesterol (TC), and triglyceride (TG) levels [2]. Currently identified common variants explain 10%–12% of the total variation in lipid levels, corresponding to ∼25% of the trait heritability [2].

Epidemiological risk factors, such as alcohol consumption, smoking, physical activity, diet and body composition are known to affect lipid levels [3]–[5]. These risk factors also show moderate to high heritabilities, and over 120 loci with genome-wide significant association have been identified (http://www.genome.gov/26525384). To better understand the biological processes modifying lipid levels, several twin studies [6]–[8] and candidate gene studies [9]–[14] have tested for interactions between genes and epidemiological risk factors.

Interactions between genes and modifiable risk factors might help us develop new lifestyle interventions targeted to susceptible individuals based on their genetic information. The effects of genetic loci and risk factors have been studied widely separately, but to date no GWA studies for interactions on lipids have been reported.

## Results

We conducted a genome-wide screen for interactions between 2.5 million genetic markers and sex, lifestyle factors (smoking and alcohol consumption), and body composition (BMI and WHR) in association to serum lipid levels (TC, TG, HDL-C, and LDL-C) in 18 population-based cohorts (max *N* = 32,225; Table S1A, Text S1). We defined interaction as a departure from a linear statistical model allowing for the additive main effects of both the SNP and the epidemiological risk factor.

**Figure 1:**
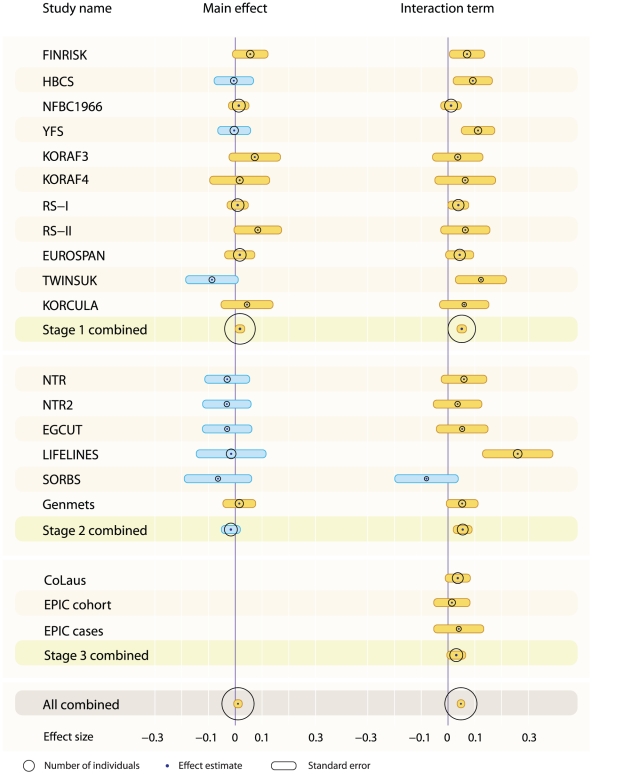
Forest plot of main and WHR interaction effect sizes of *rs6448771* on TC across the study cohorts. The circles in the plot are positioned at the effect estimates, betas, and the size corresponds to the number of individuals. The whiskers correspond to the standard errors of betas.

18 SNPs with suggestive interactions for at least one of the trait – epidemiological factor combinations (*P*-value for the interaction <10^−6^) in stage 1 analyses were taken forward to stage 2 analysis in eight additional cohorts (max *N* = 14,889; Table S1B, Text S1). In inverse variance meta-analyses combining the results from stage 1 and stage 2 (Table S2), the interaction between *rs6448771* in chromosome 4p15 and WHR on TC (Figure 1) was statistically genome-wide significant (stage 1 and 2 combined *P* = 9.08×10^−9^). This interaction was tested in stage 3 in two further cohorts (*N* = 7,813; Table S1C, Text S1), which showed an effect to the same direction. After combining results from all three stages (total *N* = 43,903), the *P*-value for interaction was 4.79×10^−9^. The association between WHR and TC was strongest for individuals carrying two G alleles of *rs6448771*, for whom a one standard deviation (sd) difference in WHR corresponds to 0.19 sd difference (confidence interval 0.13–0.25) in TC concentration, while for individuals homozygous for the A allele the difference was 0.12 sd (confidence interval 0.09–0.16) (Table S3A, Figure S1). The effect corresponds to 0.5% and 0.2% of the total variance explained in a cohort of young individuals (YFS, mean age = 37.6) and an old cohort (HBCS, mean age = 61.49), respectively. Additionally, when looking at the effect of the SNP on TC in WHR tertiles, the estimates differed in a way that the estimated SNP effect is higher for the individuals with higher WHR (Table S3B). The SNP did not have a direct effect on either TC or WHR (*P* = 0.46 and *P* = 0.51, respectively, Figure 1). The SNP *rs6448771* is located 249 kb downstream of the protocadherin 7 (*PCDH7*) gene.

Since the polymorphisms associated with complex phenotypes often influence gene expression, we examined whether individuals carrying different genotypes of *rs6448771* have variation in their transcript profiles. As WHR reflects adipose tissue function, we selected 54 individuals from Finnish dyslipidemic families with available fat biopsies and GWA data. We used linear regression to find genes that were differentially expressed in adipose tissue depending on the *rs6448771* genotype. We found two potential candidate genes with nominally significant cis-eQTL effects, *PCDH7* (*P* = 0.027, distance from the *rs6448771* 250 kb) and *CCKAR* (*P* = 0.017, distance from the SNP 4.9 Mb). The region with *CCKAR* has previously been linked with obesity [15]. Additionally, using Ingenuity software (IPA), we conducted a pathway analysis for genes with eQTL *P*-value<0.01 (both trans- and cis-eQTLs). Among other diverse IPA-defined biological functions, there was an eQTL association enrichment among genes belonging to the ‘degradation of phosphatidylcholine’ (3 genes out of 6, *P* = 6.64×10^−5^, Benjamini-Hochberg corrected *P* = 0.0138) and ‘degradation of phosphatidic acid’ (4 genes out of 8, *P* = 4.71×10^−4^, B-H corrected *P* = 0.0349) functions, which are members of broader defined IPA categories “Lipid Metabolism” and “Carbohydrate Metabolism”. These pathways were up-regulated in individuals carrying the G allele of *rs6448771*, possibly indicating a role for *rs6448771* in lipid and carbohydrate metabolism.

**Figure 2:**
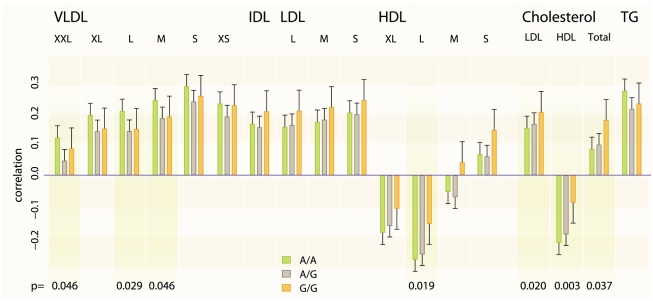
Lipoprotein subclass particle and key serum lipid concentration correlations with WHR for different genotypes of rs6448771. The height of the bar is the meta-correlation between the lipoprotein particle concentration and waist-to-hip ratio, and the whiskers correspond to standard error of the meta-correlation. The *P*-values have been taken from the interaction meta-analysis and only *P*-values<0.01 are shown in the figure. The two cohorts in which the lipid particle concentrations were measured with NMR metabonomics platform were YFS and NFBC1966 with combined number of samples of 6,500. XXL_VLDL: Chylomicrons and extremely large very low-density lipoprotein particles; XL: Very large, L: large, M: Medium, S: Small, XS: Very small; VLDL: very low-density lipoprotein; IDL: intermediate-density lipoprotein; LDL: low-density lipoprotein; HDL: High-density lipoprotein; TG: Triglycerides; TC: Total cholesterol.

The associated SNP also shows evidence for interactions with WHR on LDL-C (effect estimate for the interaction = 0.03, *P* = 0.0016) and HDL-C (effect estimate = 0.02, *P* = 0.029) in our stage 1 meta-analysis and after adjusting for TC no residual interaction effect on LDL-C and a little on HDL-C remains (*P* = 0.834 and *P* = 0.131 respectively) when testing in data subset. Therefore we tested the SNP – WHR interaction also on a range of lipoprotein subclasses measured using NMR metabonomics platform [16] available in two cohorts (NFBC1966, *N* = 4624 mean age = 31.0; YFS, *N* = 1889, mean age = 37.6). The results show that the SNP has a positive interaction effect on large HDL particle concentration (combined effect for the interaction = 0.538, *P* = 0.0186) and a negative effect on large very-low-density lipoprotein (VLDL) particles (combined effect = −0.466, *P* = 0.0291) and total triglycerides (combined effect = −0.454, *P* = 0.0343) (Figure 2).

## Discussion

Our genome-wide scan for interactions between SNP markers and traditional epidemiological risk factors in population-based random samples found a genome-wide significant locus, *rs6448771*, modifying the relationship between WHR and TC. The effect of WHR is estimated to be 64% stronger for individuals carrying two copies of the G allele than for individuals carrying two A alleles. The interaction explains around half a percent of the TC variance that is in par with the main effects of the strongest previously identified TC SNPs individually. This SNP also shows similar interaction effects on a cascade of more detailed lipid fractions suggesting broad involvement in lipid metabolism, which was also suggested by our eQTL association enrichment analysis with adipose tissue expression data.

The eQTL analysis pointed towards two potential candidate genes in the region. The first one of these was protocadherin 7 (*PCDH7*) gene, which produces a protein that is thought to function in cell-cell recognition and adhesion. The other candidate gene, cholecystokinin A receptor (*CCKAR*) regulates satiety and release of beta-endorphin and dopamine in the central and peripheral nervous system. It has been previously shown that rats with no expressed *CCKAR*s developed obesity, hyperglycemia and type 2 diabetes [17]. To test whether our eQTL finding was adipose tissue specific, we ran the eQTL analysis for *PCDH7* and *CCKAR* in another dataset with genome wide expression data from blood leukocytes (*N* = 518) available. *CCKAR* could not be tested due to its negligible expression in blood leukocytes, and no association was found for the *PCDH7* (*P*-value = 0.284) gene most likely indicating an adipose tissue specific eQTL for *PCDH7* as a function of *rs6448771*.

One interesting aspect of this study, given our large sample size, is that only one signal achieved genome-wide significance, where previously published lipid GWA studies have found close to a hundred. Although power to detect interaction is typically lower than for main effects, especially for rare exposures and SNPs, several of the exposures considered here (such as WHR, BMI, and gender) were common and available for a large proportion of the study sample. This suggests that the contribution of two-way G×E interactions to lipid levels, at least for the risk factors we examined, is rather small, or that our current measures of risk factors may not be robust enough for identifying interactions. More specific measures of both phenotypes and interacting risk factors would give better statistical power in future screens of G×E interactions.

Our findings allow us to draw several conclusions. First, to our knowledge, this is the first time an interaction between a genetic loci and a risk factor has been identified in a genome-wide scan using a stringent statistical threshold for genome-wide significance. Second, in our samples, *rs6448771* modified the relationship between WHR and TC, but was not associated with either WHR or TC alone. This observation suggests that genome-wide screens for interactions may be complementary to the current large-scale GWAS efforts for finding main effects. Third, in addition to careful harmonization of both risk factor data and phenotypes, large sample sizes are needed to identify interactions. In our study, 43,903 samples were combined to robustly identify the interaction. Our data, however, suggest that the contribution of G×E interaction using current phenotypes appears limited. Finally, from clinical point of view, the interaction may open up possibilities for targeted intervention strategies for people characterized by specific genomic profiles but more refined measures of both body-fat distribution and metabolic measures are needed to understand how their joint dynamics are modified by the newly found locus.

## Materials and Methods

### Participating studies

18 studies, with a combined sample size of over 30,000 individuals, participated in the discovery phase of this analysis; 8 studies were available for replication with over 14,000 individuals. In the discovery stage, only population-based cohorts not ascertained on the basis of phenotype, with a wide variety of well-defined epidemiological measures available, were included. In the replication datasets, the NTR cohort was selected on the basis of low risk for depression and the Genmets samples were selected for metabolic syndrome. In further replication of rs6448771, the EPIC cases were ascertained by BMI. Descriptive statistics for these populations are detailed in Table S1A (discovery), S1B (replication) and S1C (further replication). Brief descriptions of the cohorts are provided in the Text S1 section “Short descriptions of the cohorts”.

### Phenotype determination

Individuals were excluded from analysis if they were not of European descent or were receiving lipid-lowering medication at the time of sampling. TC, HDL-C, and TG concentrations were measured from serum or plasma extracted from whole blood, typically using standard enzymatic methods. LDL-C was either directly measured or estimated using the Friedewald Equation (LDL-C = TC – HDL-C – 0.45×TG for individuals with TG≤4.52 mmol/l, samples with TG level higher than 4.52 were discarded in the calculation of LDL-C) [18].

Covariates and epidemiological risk factors were ascertained at the same time that blood was drawn for lipid measurements. BMI was defined as weight in kilograms divided by the square of height in meters. Waist circumference was measured at the mid-point between the lower border of the ribs and the iliac crest; hip circumference was measured at the widest point over the buttocks. Waist-to-hip ratio was defined as the ratio of waist and hip circumferences. Alcohol consumption and smoking habits were determined via interviews and/or questionnaires. Both behaviors were coded as dichotomous (abbreviations: ALC for drinker/abstainer and SMO for current smoker/current non-smoker) and semi-quantitative traits. Semi-quantitative alcohol usage (ALCq) was based on daily consumption in grams (0: 0 g/day; 1: >0 and ≤10 g/day; 2: >10 and ≤20 g/day; 3: >20 and ≤40 g/day; 4: >40 g/day). Semi-quantitative smoking (SMOq) was assessed based on the number of cigarettes per day (0: 0 cigarettes/day; 1: >0 and ≤10 cigarettes/day; 2: >10 and ≤20 cigarettes/day; 3: >20 and ≤30 cigarettes/day; 4: >30 cigarettes/day).

### Genotyping and imputations

Affymetrix, Illumina or Perlegen arrays were used for genotyping in the discovery cohorts. Each study filtered both individuals and SNPs to ensure robustness for genetic analysis. After quality control, these data were used to impute genotypes for approximately 2.5 million autosomal SNPs based on the LD patterns observed in the HapMap 2 CEU samples. Imputed genotypes were coded as dosages, fractional values between 0 and 2 reflecting the estimated number of copies of a given allele for a given SNP for each individual. Cohort specific details concerning quality control filters, imputation reference sets and imputation software are described in Table S4.

### In silico replication

Replication cohorts utilized genome-wide imputed data, as described above, where available. Details on the genotyping methods implemented in the replication samples are described in Table S4.

### Serum NMR metabonomics, lipoprotein subclasses

Proton NMR spectroscopy was used to measure lipid, lipoprotein subclass and particle concentrations in native serum samples. NMR methods have been previously described in detail [16], [19]. Serum concentrations of total triglycerides (TG), total cholesterol (TC) together with LDL-C and HDL-C were determined. In addition, total lipid and particle concentrations in 14 lipoprotein subclasses were measured. The measurements of these subclasses have been validated against high-performance liquid chromatography [20]. The subclasses were as follows: chylomicrons and largest VLDL particles (particle diameters from approx 75 nm upwards), five different VLDL subclasses: very large VLDL (average particle diameter 64.0 nm), large VLDL (53.6 nm), medium-size VLDL (44.5 nm), small VLDL (36.8 nm), and very small VLDL (31.3 nm); intermediate-density lipoprotein (IDL) (28.6 nm); three LDL subclasses: large LDL (25.5 nm), medium-size LDL (23.0 nm), and small LDL (18.7 nm); and four HDL subclasses: very large HDL (14.3 nm), large HDL (12.1 nm), medium size HDL (10.9 nm), and small HDL (8.7 nm).

### Statistical methods

Triglyceride concentrations were natural log transformed prior to analysis. BMI and WHR were transformed to normality using inverse-normal transformation of ranks. For analyses where sex was the epidemiological variable of interest, the phenotypes were defined as the rank-inverse normal transformed residuals resulting from the regression of the lipid measurement on age and age^2^. For the other analyses, the phenotypes were defined as the inverse normal transformed residuals resulting from the regression of the lipid measurement on age, age^2^, and sex.

Associations between the transformed residuals and epidemiological risk factors/SNPs were tested using linear regression models under the assumption of an additive (allelic trend) model of genotypic effect. The models regressed phenotypes on epidemiological factor, SNP, and epidemiological factor×SNP terms
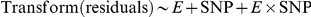
and tested if the effect for *E*×SNP was 0 using 1 df Wald tests. In family-based cohorts, linear mixed modeling was implemented to control for relatedness among samples [21]. Analysis software used by the individual cohorts is described in Table S1A and S1B.

The interaction terms from the regression analyses were meta-analyzed using inverse variance weighted fixed-effects models [22]. Prior to meta-analysis, genomic control correction factors (λ_GC_) [23], calculated from all imputed SNPs, were applied on a per-study basis to correct for residual bias possibly caused by population sub-structure. Meta-analyses were performed by two independent analysts using METAL (http://www.sph.umich.edu/csg/abecasis/Metal/index.html) and the R [24] package MetABEL (part of the GenABEL suite, http://www.genabel.org/). All results were concordant, reflecting a robust analysis. Results were selected for *in silico* replication if the meta-analysis *P*-value was less than 10^−6^. Results passing the threshold of suggestive genome-wide association (*P*-value ≤5×10^−7^) were selected for further replication by direct genotyping.

The commonly accepted genome wide level of significance (5×10^−8^) reflects the estimated testing burden of one million independent SNPs in samples of European ancestry [25]. To address the multiple testing arising from testing interactions with multiple risk factors, we set the genome wide significance threshold to 5×10^−8^/3 = 1.67×10^−8^ corresponding to three principal components explaining 97.8% of the total variation of the risk factors (Table S5).

#### Pathway analysis

The functional analyses were generated through the use of Ingenuity Pathways Analysis (Ingenuity Systems, www.ingenuity.com).” The Functional Analysis identified the biological functions and/or diseases that were most significant to the data set. Molecules which met the *P*-value cutoff of 0.01 for the rs6448771 – expression association in dataset of 54 Finnish individuals with both genotype and adipose tissue expression data, and were associated with biological functions and/or diseases in Ingenuity's Knowledge Base were considered for the analysis. Right-tailed Fisher's exact test was used to calculate a *P*-value determining the probability that each biological function and/or disease assigned to that data set is due to chance alone and Benjamini-Hochberg multiple test correction [26] was applied.

## Supporting Information

Figure S1 Effect of waist-to-hip ratio on total cholesterol as a function of *rs6448771* genotypes. The bars in the plot are the effect estimates from three meta-analyzed linear models where total cholesterol (TC) has been explained using waist-to-hip ratio (WHR). The analyses were ran in three strata based on the *rs6448771* genotypes. The whiskers in the plot correspond to the confidence intervals of the effect estimates.
(DOC)

Table S1 Cohort characteristics. The number of study subjects with available phenotype and genotype (lower line) and summary statistics (upper line) for every cohort and trait. For continuous traits mean (standard deviation) is presented. For dichotomous traits number of individuals with phenotype present (%) is presented. TC: total cholesterol (mmol/l); HDL-C: high-density lipoprotein cholesterol (mmol/l); LDL-C: low-density lipoprotein cholesterol (mmol/l); TG: triglycerides (mmol/l); BMI: body-mass index; WHR: waist-to-hip ratio; NA: not available.
(DOC)

Table S2 Loci having *P*-value<1×10^−6^ in Stage 1 analyses and replication of the SNPs. Best SNP per locus having *P*-value<1×10^−6^ in the Stage 1 analysis combining 19 cohorts. The bolded number is the genome-wide significant *P*-value. *N*: number of individuals; *SE*: standard error of the effect estimate, Beta; LDL-C: low-density lipoprotein cholesterol; TC: total cholesterol; TG: triglycerides; HDL-C: high-density lipoprotein cholesterol; ALC: alcohol usage (drinker/abstainer); WHR: waist-to-hip ratio; BMI: body mass index; SMO: smoking (current/not);; SMOq: semi-quantitative smoking (0: 0 cigarettes/day; 1: >0 and ≤10 cigarettes/day; 2: >10 and ≤20 cigarettes/day; 3: >20 and ≤30 cigarettes/day; 4: >30 cigarettes/day); ALCq: semi-quantitative alcohol (0: 0 g/day; 1: >0 and ≤10 g/day; 2: >10 and ≤20 g/day; 3: >20 and ≤40 g/day; 4: >40 g/day).
(DOC)

Table S3 Effect of *rs6448771* on total cholesterol (TC) by waist-to-hip ratio (WHR) tertiles and effect of WHR on TC by SNP genotype classes. Section A shows the combined effect of waist-to-hip ratio (WHR) on total cholesterol (TC) stratified by the *rs6448771* genotype class from five Finnish cohorts (FINRISK, NFBC1966, YFS, Genmets and HBCS, combined number of individuals is 12,782) and section B shows the combined effect of the SNP on TC stratified by WHR tertiles from the same cohorts. The limit values for the waist-to-hip ratio (WHR) tertiles have been calculated using WHR values from all five datasets. Both analyses were ran using untransformed and standardized scales and were adjusted with age, age^2^ and sex. Beta: effect estimate; *CI*: confidence interval.
(DOC)

Table S4 Details of GWA data in discovery and replication cohorts. QC: quality control; MAF: minor allele frequency; HWE: Hardy-Weinberg equilibrium.
(DOC)

Table S5 Proportions of variance explained by principal components. Principal components analysis (PCA) was run for the seven risk factors used in the screening. PC: Principal Component.
(DOC)

Text S1 Short descriptions of the cohorts and a full list of acknowledgements.
(DOC)
