# Supplementary figures and images for: A Genome-Wide Screen for Interactions Reveals a New Locus on 4p15 Modifying the Effect of Waist-to-Hip Ratio on Total Cholesterol
Source: PLoS Genet. 2011 Oct 20;7(10):e1002333. doi: 10.1371/journal.pgen.1002333 (PMC3197672; doi:10.1371/journal.pgen.1002333)

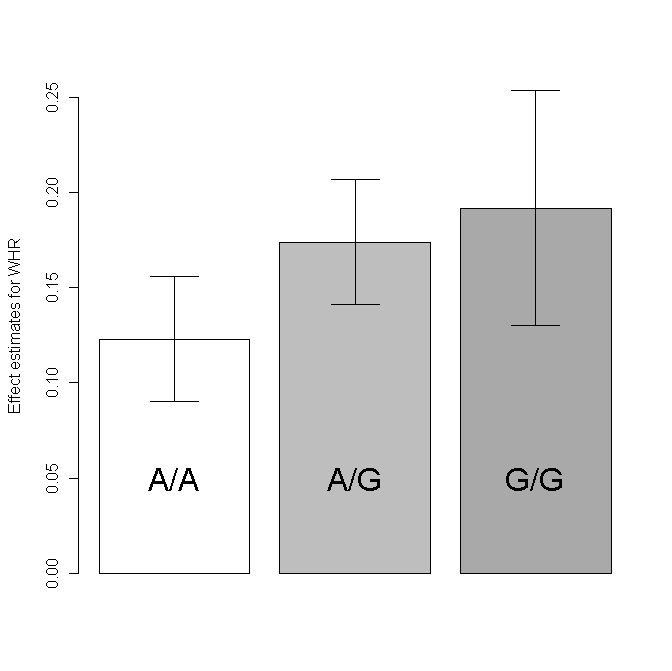

Supplement: Figure S1 — Effect of waist-to-hip ratio on total cholesterol as a function of rs6448771 genotypes. The bars in the plot are the effect estimates from three meta-analyzed linear models where total cholesterol (TC) has been explained using waist-to-hip ratio (WHR). The analyses were ran in three strata based on the rs6448771 genotypes. The whiskers in the plot correspond to the confidence intervals of the effect estimates. (DOC) [file pgen.1002333.s001.doc]
