# Supplementary material for: A Genome-Wide Screen for Interactions Reveals a New Locus on 4p15 Modifying the Effect of Waist-to-Hip Ratio on Total Cholesterol
Source: PLoS Genet. 2011 Oct 20;7(10):e1002333. doi: 10.1371/journal.pgen.1002333 (PMC3197672; doi:10.1371/journal.pgen.1002333)
Supplement: Table S1 — Cohort characteristics. The number of study subjects with available phenotype and genotype (lower line) and summary statistics (upper line) for every cohort and trait. For continuous traits mean (standard deviation) is presented. For dichotomous traits number of individuals with phenotype present (%) is presented. TC: total cholesterol (mmol/l); HDL-C: high-density lipoprotein cholesterol (mmol/l); LDL-C: low-density lipoprotein cholesterol (mmol/l); TG: triglycerides (mmol/l); BMI: body-mass index; WHR: waist-to-hip ratio; NA: not available. (DOC) [file pgen.1002333.s002.doc]

A: Stage 1 cohorts

| Population |  | TC | HDL-C | LDL-C | TG | Age | Male Gender | BMI | WHR | Smoking | Alcohol |
| --- | --- | --- | --- | --- | --- | --- | --- | --- | --- | --- | --- |
| ATFS |  | 5.35 (1.13) 4948 | 1.50 (0.41) 4896 | 3.10 (0.97) 4769 | 1.66 (1.13) 4947 | 36.30 (16.1)  4954 | 1789 (36.0) | 24.17 (5.10) 3758 | NA | 718 (29.0) | 2234 (82.0) |
| DKTWIN |  | 5.48 (1.24)  168 | 1.62 (0.44)  140 | 3.19 (1.00)  138 | 1.19 (0.46)  140 | 44.06 (19.17)  169 | 0 (0.0) | 23.62 (3.71)  166 | NA | NA | NA |
| ERF |  | 5.65 (1.09) 1898 | 1.30 (0.36) 1898 | 3.81 (0.97) 1898 | 1.28 (0.70) 1898 | 46.94 (14.39) 1898 | 809 (42.6) | 26.57 (4.54) 1898 | 0.86 (0.10) 1898 | 734 (38.7) | 1297 (68.3) |
| FINRISK |  | 5.54 (1.01) 1896 | 1.44 (0.40) 1896 | 3.43 (0.89) 1867 | 1.50 (0.95) 1896 | 55.59 (12.2) 1896 | 1015 (53.5) | 26.99 (4.44) 1884 | 0.91 (0.09) 1893 | 579 (64.4) | NA |
| FINTWIN |  | 5.37 (0.95)  138 | 1.61 (0.46)  138 | 3.47 (0.98)  138 | 1.10 (0.53)  39 | 60.29 (15.78)  138 | 0 (0.0) | 24.85 (3.68)  125 | 0.85 (0.06)  122 | 15 (13.0) | NA |
| HBCS |  | 5.94 (1.04) 1726 | 1.61 (0.43) 1726 | 3.66 (0.88) 1691 | 1.53 (1.1)  1726 | 61.49 (2.93)  1728 | 737 (42.7) | 27.65 (4.65) 1725 | 0.92 (0.09) 1723 | 353 (43.7) | 1301 (92.0) |
| KORAF3 |  | 5.82 (1.01) 1405 | 1.52 (0.44) 1405 | 3.45 (0.83) 1405 | 1.93 (1.36) 1405 | 62.52 (10.09) 1644 | 813 (49.45) | 28.14 (4.48) 1636 | 0.90 (0.08) 1639 | 198 (12.06) | 1158 (70.61) |
| KORAF4 |  | 5.84 (1.00) 1515 | 1.48 (0.38) 1515 | 3.72 (0.89) 1515 | 1.48 (1.06) 1515 | 60.91 (8.86) 1814 | 884 (48.7) | 28.16 (4.80) 1808 | 0.89 (0.09) 1810 | 266 (14.8) | 1272 (70.2) |
| KORCULA |  | 5.96 (1.23)  886 | 1.46 (0.35)  886 | 3.83 (1.04)  886 | 1.43 (0.78)  886 | 56.22 (14.02)  886 | 319 (36.0) | 27.92 (4.06)  886 | 0.90 (0.09)  886 | 201 (22.7) | 567 (64.0) |
| YFS |  | 5.03 (0.90) 2023 | 1.34 (0.32) 2011 | 3.08 (0.78) 1984 | 1.38 (0.88) 2022 | 37.6 (5.03)  2443 | 1320 (54.0) | 25.9 (4.66)  1987 | 0.88 (0.09) 1999 | 372 (18.4) | 1486 (74.0) |
| MICROS |  | 5.88 (1.21) 1037 | 1.69 (0.37) 1037 | 3.55 (1.11) 1037 | 1.37 (0.78) 1037 | 44.39 (15.78) 1037 | 451 (43.5) | 25.50 (4.61) 1037 | 0.90 (0.10) 1037 | 467 (45.0) | 803 (77.4) |
| NFBC1966 |  | 5.08 (1.00) 5262 | 1.56 (0.38) 5263 | 3.01 (0.89) 5262 | 1.19 (0.70) 5258 | 31.00 (0)  5363 | 2569 (48.1) | 24.6 (4.03)  5239 | 0.86 (0.08) 5072 | 2113 (40.7) | 3705 (91.2) |
| NLDTWIN |  | 4.85 (0.97)  287 | 1.54 (0.38)  286 | 2.82 (0.93)  287 | 1.07 (0.49)  287 | 32.2 (11.34)  287 | 0 (0.0) | 21.83 (3.00)  240 | NA | NA | NA |
| NSPHS |  | 5.90 (1.35)  561 | 1.60 (0.39)  561 | 3.58 (1.09)  561 | 2.22 (1.57)  561 | 44.99 (20.29)  561 | 257 (45.8) | 26.17 (4.74)  561 | NA | 85 (15.2) | 266 (47.4) |
| ORCADES |  | 5.88 (1.09)  633 | 1.70 (0.40)  633 | 3.64 (1.04)  633 | 1.29 (0.62)  633 | 51.92 (15.67)  633 | 288 (45.5) | 27.46 (4.87)  633 | 0.90 (0.09)  633 | 57 (9.0) | 558 (88.2) |
| RSI |  | 6.60 (1.21)  5874 | 1.34 (0.37)  5849 | 3.75 (0.88)  2302 | 1.54 (0.75)  2359 | 69.4 (9.1)  5974 | 2359 (40.5) | 26.30 (3.69)  5745 | 0.91 (0.09)  5468 | 3764 (64.8) | 3663 (79.5) |
| RSII |  | 5.86 (0.97) 1628 | 1.39 (0.37) 1628 | 3.79 (0.89) 1628 | 1.54 (0.78) 1628 | 64.71 (8.15)  1628 | 732 (45.0) | NA | NA | 318 (19.5) | 298 (53.5) |
| SWETWIN |  | 6.04 (1.09)  302 | 1.52 (0.41)  302 | 3.88 (0.97)  297 | 1.47 (0.87)  302 | 71.85 (5.87)  302 | 0 (0.0) | 24.71 (3.66)  298 | NA | NA | NA |
| TWINSUK |  | 5.39 (1.22) 1349 | 1.55 (0.43) 1349 | 3.38 (1.00)  974 | 1.05 (0.58) 1336 | 50.3 (13.00)  1349 | 0 (0) | 25.04 (4.70) 1343 | 0.77 (0.05)  968 | 496 (58.2) | 696 (73.0) |
| VIS |  | 5.10 (1.00)  774 | 1.11 (0.15)  774 | 3.22 (0.93)  774 | 1.69 (0.90)  774 | 56.49 (15.34)  774 | 325 (42.0) | 27.32 (4.18)  774 | 0.93 (0.09)  774 | 216 (27.9) | 205 (26.5) |

B: Stage 2 cohorts

| Population |  | TC | HDL-C | LDL-C | TG | Age | Male Gender | BMI | WHR | Smoking | Alcohol |
| --- | --- | --- | --- | --- | --- | --- | --- | --- | --- | --- | --- |
| EGCUT |  | 5.26 (1.11)  847 | 1.54 (0.44)  847 | 3.37 (1.03)  847 | 1.52 (0.92)  847 | 37.1 (15.5)  847 | 409 (48.2) | 25.0 (4.43)  847 | 0.83 (0.09)  847 | 217 (25.6) | 798 (94.2) |
| LIFELINES |  | 5.38 (0.97) 2981 | 1.47 (0.39) 2980 | 3.55 (0.88) 2981 | 1.30 (0.83) 2981 | 54.47 (9.65) 2981 | 1179 (39.6) | 26.46 (4.13) 2981 | 0.92 (0.08) 2980 | 572 (19.2) | 2401 (82.8) |
| NTR |  | 5.17 (1.08) 1475 | 1.41 (0.39) 1475 | 3.16 (0.95) 1475 | 1.32 (0.72) 1475 | 44.65 (13.80) 1475 | 532 (36.1) | 24.95 (3.79) 1466 | 0.83 (0.09) 1464 | 290 (19.8) | 1125 (81.8) |
| NTR2 |  | 5.31 (1.05) 1069 | 1.43 (0.40) 1069 | 3.24 (0.93) 1069 | 1.39 (0.80) 1069 | 48.68 (13.96) 1069 | 400 (37.4) | 25.53 (3.99) 1052 | 0.84 (0.09) 1052 | 217 (20.4) | 825 (79.5) |
| PREVEND |  | 5.66 (1.10)  3942 | 1.31 (0.40) 3884 | 3.69 (1.03) 3801 | 1.44 (0.98) 3762 | 49.59 (12.47) 3969 | 2027 (51.1) | 26.14 (4.28) 3938 | 0.89 (0.10) 3938 | 1420 (35.9) | 2994 (75.9) |
| RSIII |  | 5.69 (1.01) 1804 | 1.48 (0.45) 1804 | 3.56 (0.91) 1804 | 1.47 (0.97) 1804 | 55.69 (5.72) 1804 | 746 (41.4) | 27.49 (4.67) 1804 | NA | 401 (22.2) | 1634 (90.6) |
| SORBS |  | 5.34 (1.05) 829 | 1.64 (0.40) 829 | 3.40 (0.98) 828 | 1.11 (1.69) 829 | 47.7 (16.35) 932 | 384 (40.9) | 26.9 (4.91) 921 | 0.87 (0.10) 936 | 191 (20.6) | 508 (54.5) |
| Genmets |  | 6.02 (1.08) 2124 | 1.33 (0.37) 2124 | 3.77 (1.03) 2124 | 1.63 (0.98) 2124 | 50.74 (11.11) 2124 | 1036 (48.8) | 27.3 (4.51) 2123 | 0.92 (0.08) 2122 | 519 (24.5) | 1839 (89.7) |

C: Stage 3 cohorts

| Population |  | TC | Age | Male Gender | WHR |
| --- | --- | --- | --- | --- | --- |
| CoLaus |  | 5.64 (1.04) 4791 | 52.38 (10.56) 4791 | 2184 (45.59) 4791 | 0.87 (0.08) 4791 |
| EPIC cohort |  | 6.18 (1.14)  2005 | 59.35 (8.97)  2005 | 932 (46.5)  2005 | 0.86 (0.09)  2005 |
| EPIC cases |  | 6.36 (1.17)  1017 | 59.94 (8.77)  1017 | 439 (43.2)  1017 | 0.90 (0.10)  1017 |
