# Supplementary material for: A Genome-Wide Screen for Interactions Reveals a New Locus on 4p15 Modifying the Effect of Waist-to-Hip Ratio on Total Cholesterol
Source: PLoS Genet. 2011 Oct 20;7(10):e1002333. doi: 10.1371/journal.pgen.1002333 (PMC3197672; doi:10.1371/journal.pgen.1002333)
Supplement: Table S2 — Loci having P-value<1×10^−6^ in Stage 1 analyses and replication of the SNPs. Best SNP per locus having P-value<1×10^−6^ in the Stage 1 analysis combining 19 cohorts. The bolded number is the genome-wide significant P-value. N: number of individuals; SE: standard error of the effect estimate, Beta; LDL-C: low-density lipoprotein cholesterol; TC: total cholesterol; TG: triglycerides; HDL-C: high-density lipoprotein cholesterol; ALC: alcohol usage (drinker/abstainer); WHR: waist-to-hip ratio; BMI: body mass index; SMO: smoking (current/not);; SMOq: semi-quantitative smoking (0: 0 cigarettes/day; 1: >0 and ≤10 cigarettes/day; 2: >10 and ≤20 cigarettes/day; 3: >20 and ≤30 cigarettes/day; 4: >30 cigarettes/day); ALCq: semi-quantitative alcohol (0: 0 g/day; 1: >0 and ≤10 g/day; 2: >10 and ≤20 g/day; 3: >20 and ≤40 g/day; 4: >40 g/day). (DOC) [file pgen.1002333.s003.doc]

| Trait | Interaction | SNP | Chromosome | Position | Gene(s) |  | Effect allele | Other allele | Effect allele frequency | *N* | Initial analyses  (Stage 1)  Beta  (*SE*) | Initial analysis  *P*-value | *In silico* replication (Stage 2) Beta  (*SE*) | Further replication (Stage 3)  Beta  (*SE*) | Combined *N* | Combined  *P*-value |
| --- | --- | --- | --- | --- | --- | --- | --- | --- | --- | --- | --- | --- | --- | --- | --- | --- |
| LDL-C | ALC | *rs1862101* | 2 | 15,761,291 | *NBAS/NAG/DDX1/*  *N-cym/MYCN* |  | C | T | 0.499 | 21,539 | -0.125 (0.025) | 4.60 × 10^-7^ | -0.005 (0.031) |  | 35,993 | 5.80 × 10^-5^ |
| TC | ALC | *rs1862101* | 2 | 15,761,291 | *NBAS/NAG/DDX1/*  *N-cym/MYCN* |  | C | T | 0.501 | 24,242 | -0.117 (0.023) | 5.13 × 10^-7^ | 0.005 (0.031) |  | 38,863 | 9.16 × 10^-5^ |
| TG | WHR | *rs9856132* | 3 | 63,360,423 | *SYNPR* |  | C | G | 0.470 | 23,520 | -0.049 (0.010) | 2.49 × 10^-7^ | 0.007 (0.013) |  | 36,411 | 1.09 × 10^-4^ |
| HDL-C | WHR | *rs775713* | 3 | 77,699,909 | *ROBO2* |  | C | G | 0.489 | 26,769 | -0.044 (0.009) | 9.57 × 10^-7^ | 0.005 (0.013) |  | 35,548 | 1.49 × 10^-4^ |
| TC | BMI | *rs4679788* | 3 | 158,437,085 | *LEKR1/CCNL1/UNQ530/*  *VEPH1/PTX3* |  | C | T | 0.278 | 32,225 | 0.043 (0.009) | 4.49 × 10^-7^ | 0.026 (0.015) |  | 43,336 | 2.93 × 10^-7^ |
| TC | WHR | *rs6448771* | 4 | 31,006,716 | *PCDH7* |  | G | A | 0.335 | 26,801 | 0.049 (0.010) | 5.38 × 10^-7^ | 0.052 (0.018) | 0.028  (0.018) | 43,903 | **4.79 × 10^-9^** |
| TC | BMI | *rs7717939* | 5 | 2,277,557 | *none* |  | C | G | 0.444 | 32,225 | 0.041 (0.008) | 9.34 × 10^-7^ | -0.003 (0.013) |  | 47,105 | 6.91 × 10^-5^ |
| TC | SMO | *rs2328534* | 6 | 144,307,786 | *PHACTR2/LTV1/FAM164B/*  *PALGL1/*  *HYMAI/STX11* |  | C | G | 0.748 | 28,183 | -0.147 (0.029) | 5.03 × 10^-7^ | 0.069 (0.050) |  | 43,072 | 2.67 × 10^-4^ |
| TG | SEX | *rs2304327* | 7 | 43,485,862 | *HECW1/STK17A* |  | C | T | 0.149 | 28,098 | 0.125 (0.025) | 3.58 × 10^-7^ | 0.008 (0.035) |  | 42,850 | 1.81 × 10^-5^ |
| TG | SMO | *rs6987225* | 8 | 99,044,848 | *MATN2* |  | C | A | 0.061 | 24,743 | 0.231 (0.043) | 5.66 × 10^-8^ | 0.043 (0.066) |  | 39,453 | 9.17 × 10^-7^ |
| LDL-C | SMOq | *rs7894832* | 10 | 36,682,081 | *CCNY/GJD4/FZD8/ANKRD30A* |  | G | A | 0.827 | 15,674 | 0.090 (0.018) | 8.38 × 10^-7^ | 0.006 (0.034) |  | 20,565 | 9.88 × 10^-6^ |
| HDL-C | WHR | *rs12360783* | 11 | 10,141,941 | *SBF2/ADM* |  | C | T | 0.275 | 26,769 | -0.049 (0.010) | 6.21 × 10^-7^ | 0.006 (0.014) |  | 39,778 | 1.48 × 10^-4^ |
| LDL-C | ALC | *rs471071* | 13 | 100,742,810 | *NALCN* |  | C | T | 0.105 | 21,539 | -0.227 (0.041) | 2.96 × 10^-8^ | 0.026 (0.058) |  | 35,993 | 1.91 × 10^-5^ |
| TG | ALCq | *rs7207298* | 17 | 22,019,254 | *FAM27L* |  | C | T | 0.324 | 16,813 | 0.054 (0.011) | 8.94 × 10^-7^ | -0.032 (0.023) |  | 19,687 | 1.48 × 10^-4^ |
| HDL-C | SMOq | *rs17676191* | 17 | 35,203,450 | *NEUROD2/PPP1R1B/STARD3/TCAP/PNMT/*  *CAB2/HER-2/ERBB2/GRB7/IKZF3/*  *FPBP2/GSDMB/ORMDL3/PSMD3/*  *C3F3/MED24* |  | G | A | 0.084 | 19,120 | 0.118 (0.024) | 5.00 × 10^-7^ | -0.010 (0.036) |  | 25,866 | 4.70 × 10^-5^ |
| LDL-C | SMO | *rs11077764* | 17 | 70,299,279 | *CD300-cluster/RAB37/NKIR/*  *NHERF/NAT9/TMEM104/GRIN2C/*  *FDXR/FADS6/USH1G/OTOP2&3/*  *CDRL2/ICT1/ATP5/KCTD2* |  | C | T | 0.526 | 23,173 | 0.112 (0.023) | 6.94 × 10^-7^ | -0.021 (0.031) |  | 37,072 | 3.05 × 10^-4^ |
| HDL-C | SEX | *rs17271418* | 19 | 52,677,032 | *BBC3/CCDC9/C5AR1/GPR77/*  *DHX34/MEIS3/SLC8A2/KPTN/*  *NAPA/ZNF541/GLTSCR1/EHD2* |  | G | A | 0.910 | 31,464 | -0.151 (0.030) | 4.44 × 10^-7^ | 0.008 (0.054) |  | 46,333 | 1.43 × 10^-5^ |
| HDL-C | ALCq | *rs16981145* | 20 | 19,738,550 | *SLC24A3/RIN2* |  | C | G | 0.080 | 19,207 | 0.081 (0.016) | 4.93 × 10^-7^ | 0.073 (0.046) |  | 22,081 | 1.34 × 10^-7^ |
| HDL-C | SEX | *rs1297152* | 21 | 14,615,347 | *LIPI/RBM11/ABCC13/HSPA13/*  *SAMSN1* |  | C | T | 0.430 | 31,464 | -0.076 (0.016) | 8.41 × 10^-7^ | 0.004 (0.024) |  | 46,334 | 4.84 × 10^-5^ |
