# Supplementary material for: A Genome-Wide Screen for Interactions Reveals a New Locus on 4p15 Modifying the Effect of Waist-to-Hip Ratio on Total Cholesterol
Source: PLoS Genet. 2011 Oct 20;7(10):e1002333. doi: 10.1371/journal.pgen.1002333 (PMC3197672; doi:10.1371/journal.pgen.1002333)
Supplement: Table S3 — Effect of rs6448771 on total cholesterol (TC) by waist-to-hip ratio (WHR) tertiles and effect of WHR on TC by SNP genotype classes. Section A shows the combined effect of waist-to-hip ratio (WHR) on total cholesterol (TC) stratified by the rs6448771 genotype class from five Finnish cohorts (FINRISK, NFBC1966, YFS, Genmets and HBCS, combined number of individuals is 12,782) and section B shows the combined effect of the SNP on TC stratified by WHR tertiles from the same cohorts. The limit values for the waist-to-hip ratio (WHR) tertiles have been calculated using WHR values from all five datasets. Both analyses were ran using untransformed and standardized scales and were adjusted with age, age2 and sex. Beta: effect estimate; CI: confidence interval. (DOC) [file pgen.1002333.s004.doc]

A

| Effect of raw WHR on raw TC by rs6448771 classes |  | | | Effect of standardized WHR on standardized TC by rs6448771 classes |  | | |
| --- | --- | --- | --- | --- | --- | --- | --- |
|  | Beta | *CI*-95 lower | *CI*-95 upper |  | Beta | *CI*-95 lower | *CI*-95 upper |
| AA | 1.373 | 0.995 | 1.751 | AA | 0.123 | 0.090 | 0.156 |
| AG | 2.005 | 1.626 | 2.384 | AG | 0.174 | 0.141 | 0.207 |
| GG | 2.186 | 1.482 | 2.889 | GG | 0.192 | 0.130 | 0.254 |

B

| SNP effect on raw TC by WHR tertiles |  | | | SNP effect on standardized TC by WHR tertiles |  | | |
| --- | --- | --- | --- | --- | --- | --- | --- |
|  | Beta | *CI*-95 lower | *CI*-95 upper |  | Beta | *CI*-95 lower | *CI*-95 upper |
| WHR <0.84375 | -0.031 | -0.069 | 0.008 | WHR <0.84375 | -0.030 | -0.068 | 0.009 |
| 0.84375< WHR <0.92891 | -0.011 | -0.053 | 0.031 | 0.84375< WHR <0.92891 | -0.011 | -0.053 | 0.031 |
| WHR >0.92891 | 0.083 | 0.036 | 0.129 | WHR >0.92891 | 0.082 | 0.036 | 0.127 |
