# Supplementary material for: A Genome-Wide Screen for Interactions Reveals a New Locus on 4p15 Modifying the Effect of Waist-to-Hip Ratio on Total Cholesterol
Source: PLoS Genet. 2011 Oct 20;7(10):e1002333. doi: 10.1371/journal.pgen.1002333 (PMC3197672; doi:10.1371/journal.pgen.1002333)
Supplement: Table S4 — Details of GWA data in discovery and replication cohorts. QC: quality control; MAF: minor allele frequency; HWE: Hardy-Weinberg equilibrium. (DOC) [file pgen.1002333.s005.doc]

| Population |  | Genotyping  Platform | Calling Software | Imputation  QC Filters |  |  | Imputation Reference | Imputation Software | Analysis Software | Adjustment for Relatedness |
| --- | --- | --- | --- | --- | --- | --- | --- | --- | --- | --- |
|  |  |  |  | Call Rate | MAF | HWE  *P*-value |  |  |  |  |
| ATFS |  | Illumina 370/610 | BeadStudio | < 95% | < 1% | < 10^-5^ | B36rel22 | Mach v 1.0.15 | ProbABEL | NA |
| DKTWIN |  | Illumina 318 | BeadStudio | < 99% | < 1% | < 10^-6^ | B36rel22 | Mach v 1.0.16 | ProbABEL | NA |
| ERF |  | Illumina 318/370 | BeadStudio | < 98% | < 1% | < 10^-6^ | B36rel22 | Mach v 1.0.15 | GenABEL/ ProbABEL | mmscore |
| FINRISK |  | Illumina 610 | Illuminus | < 95% | < 1% | < 10^-6^ | B36rel22 | Mach v 1.0.16 | ProbABEL | NA |
| FINTWIN |  | Illumina 318 | BeadStudio | < 99% | < 1% | < 10^-6^ | B36rel22 | Mach v 1.0.16 | ProbABEL | NA |
| HBCS |  | Illumina 670 | Illuminus | < 95% | < 1% | < 10^-6^ | B36rel22 | Mach v 1.0.16 | ProbABEL | NA |
| KORAF3 |  | Affymetrix 500 | BRLMM | < 95% | < 1% | < 10^-5^ | B35rel21 | Mach1 v1.010 | ProbABEL | NA |
| KORAF4 |  | Affymetrix 6.0 | Birdseed | < 93% | < 1% | < 10^-5^ | B36rel22 | Mach v 1.0.16 | ProbABEL | NA |
| KORCULA |  | Illumina 370 | BeadStudio | < 98% | < 1% | < 10^-6^ | B36rel22 | Mach v 1.0.15 | GenABEL/ ProbABEL | mmscore |
| YFS |  | Illumina 670 | Illuminus | < 95% | < 1% | < 10^-6^ | B36rel22 | Mach v 1.0.16 | ProbABEL | NA |
| MICROS |  | Illumina HumHap 300v2 | BeadStudio | < 98% | < 1% | < 10^-6^ | B36rel22 | Mach v 1.0.16 | GenABEL/ ProbABEL | mmscore |
| NFBC1966 |  | Illumina 370 | Illuminus | < 95% | < 1% | < 10^-6^ | B36rel22 | Mach v 1.0.16 | ProbABEL | NA |
| NLDTWIN |  | Illumina 318 | BeadStudio | < 99% | < 1% | < 10^-6^ | B36rel22 | Mach v 1.0.16 | ProbABEL | NA |
| NSPHS |  | Illumina 318 | BeadStudio | < 98% | < 1% | < 10^-6^ | B36rel22 | Mach v 1.0.15 | GenABEL/ ProbABEL | mmscore |
| ORCADES |  | Illumina 370/ HumHap 300v2 | BeadStudio | < 98% | < 1% | < 10^-6^ | B36rel22 | Mach 1.0 ML | GenABEL/ ProbABEL | mmscore |
| RSI |  | Illumina 550 | BeadStudio | < 98% | < 1% | < 10^-6^ | B36rel22 | Mach v 1.0.15 | ProbABEL | NA |
| RSII |  | Illumina 550/610 | GenomeStudio | < 98% | < 1% | < 10^-6^ | B36rel22 | Mach v 1.0.16 | ProbABEL | NA |
| SWETWIN |  | Illumina 318 | BeadStudio | < 99% | < 1% | < 10^-6^ | B36rel22 | Mach v 1.0.16 | ProbABEL | NA |
| TWINSUK |  | Illumina 300/550/610 | Illuminas | < 95% | < 1% | < 10^-4^ | B36rel22 | Impute v 0.3.2 | SNPTEST v 1.1.4 | Huber-White |
| VIS |  | Illumina 318 | BeadStudio | < 98% | < 1% | < 10^-6^ | B36rel22 | Mach v 1.0.15 | GenABEL/ ProbABEL | mmscore |
| EGCUT |  | Illumina 370CNV /OmniExpress | Genomestudio | < 95% | < 1% | < 10^-6^ | B36re22 | Impute v1.0 | PLINK/GenABEL/ProbABEL | NA |
| LIFELINES |  | Illumina HumanCytoSNP-12 | Genomestudio | <95% | <1% | <10^-5^ | B36R23A | BEAGLE v3.1.0 | ProbABEL_0.1-3_win | NA |
| NTR |  | PERLEGEN-AFFY600K | Perlegen proprietary | < 95% | < 1% | < 10^-5^ | B36re22 | Impute 0.5.0 | Quicktest/PLINK | NA |
| NTR2 |  | ILLUMINA660 | BEADSTUDIO | < 95% | < 1% | < 10^-5^ | B36re22 | Impute 0.5.0 | PLINK | NA |
| PREVEND |  | Illumina HumanCytoSNP-12 | Genomestudio | <98% | <1% | <10^-5^ | B36R23A | BEAGLE v3.1.0 | ProbABEL_0.1-3_win | NA |
| RSIII |  | Illumina 610K | Genome Studio | < 98% | < 1% | < 10^-6^ | B36rel22 | Mach v 1.0.16 | ProbABEL | NA |
| SORBS |  | Affymetrix 500K and Affymetrix 6.0 | BRLMM Birdseed | <95% | <1% | <10^-4^ | B35 | Impute 1.0.0 | ProbABEL 0.1-9c | mmscore |
| Genmets |  | Illumina 610K | Illuminus | < 95% | < 1% | < 10^-6^ | B36rel22 | Mach v 1.0.16 | ProbABEL | NA |
| CoLaus |  | Affymetrix 500K | BRLMM | < 70% | <1% | <1E-7 | B35rel21 | Impute 0.2.0 | Matlab | NA |
| EPIC cohort |  | Infinium HumanHap300 | GenomeStudio | NA | NA | NA | NA | NA | PLINK | NA |
| EPIC cases |  | Infinium HumanHap300 | GenomeStudio | NA | NA | NA | NA | NA | PLINK | NA |
