# Supplementary material for: A Genome-Wide Screen for Interactions Reveals a New Locus on 4p15 Modifying the Effect of Waist-to-Hip Ratio on Total Cholesterol
Source: PLoS Genet. 2011 Oct 20;7(10):e1002333. doi: 10.1371/journal.pgen.1002333 (PMC3197672; doi:10.1371/journal.pgen.1002333)
Supplement: Table S5 — Proportions of variance explained by principal components. Principal components analysis (PCA) was run for the seven risk factors used in the screening. PC: Principal Component. (DOC) [file pgen.1002333.s006.doc]

|  | PC1 | PC2 | PC3 | PC4 | PC5 | PC6 | PC7 |
| --- | --- | --- | --- | --- | --- | --- | --- |
| Standard deviation | 3.983 | 1.102 | 0.800 | 0.456 | 0.378 | 0.195 | 0.050 |
| Proportion of Variance | 0.876 | 0.067 | 0.035 | 0.011 | 0.008 | 0.002 | 0.0001 |
| Cumulative Proportion | 0.876 | 0.943 | 0.978 | 0.990 | 0.998 | 0.9999 | 1.00000 |
