# Supplementary material for: A Genome-Wide Screen for Interactions Reveals a New Locus on 4p15 Modifying the Effect of Waist-to-Hip Ratio on Total Cholesterol
Source: PLoS Genet. 2011 Oct 20;7(10):e1002333. doi: 10.1371/journal.pgen.1002333 (PMC3197672; doi:10.1371/journal.pgen.1002333)
Supplement: Text S1 — Short descriptions of the cohorts and a full list of acknowledgements. (DOC) [file pgen.1002333.s007.doc]

**Short descriptions of the cohorts**

**ATFS:** Lipid traits were measured in serum samples from adolescent twins and their siblings, and from adult twins and their first-degree relatives, and genome-wide SNP markers were genotyped. For this analysis, one person from each family was used (i.e. unrelated subjects only). The study participants comprise: (1) Adolescent twins and their non-twin siblings living in south-east Queensland (Australia) who had participated in the Brisbane Longitudinal Twin study [27]. A total of 1,041 families (mean age of 13.5 years) were genotyped. (2) Adult twins consisting of twins and their family members who participated in studies of: (i) alcohol and nicotine dependence and metabolic risk for alcoholic liver disease [28]; (ii) Anxiety and Depression [29]; and (iii) Endometriosis [30]. A total of 3,913 (mean age of 42.3 years) families were genotyped. Where multiple measurements of the same trait in an individual were available, the average of the values was used. For each of these studies, participants (and, for subjects aged < 18 years, their parents) gave informed consent to the questionnaire, interview, and blood collection, and all studies were approved by the QIMR Human Research Ethics Committee. Serum cholesterol, HDL cholesterol and triglycerides were measured using Roche methods on a Roche 917 or Modular P analyzer (Roche Diagnostics, Basel, Switzerland) and LDL cholesterol was calculated using the Friedewald equation.

**EUROSPAN:** The European Special Populations Network (EUROSPAN) consortium consists of 5 population samples:

ERF: The Erasmus Rucphen Family study [31] (ERF) is comprised of a family-based cohort embedded in the Genetic Research in Isolated Populations (GRIP) program in the southwest of the Netherlands. The aim of this program is to identify genetic risk factors for the development of complex disorders. In ERF, twenty-two families that had a minimum of five children baptized in the community church between 1850 and 1900 were identified with the help of detailed genealogical records. All living descendants of these couples, and their spouses, were invited to take part in the study. Comprehensive interviews, questionnaires, and examinations were completed at a research center in the area and blood was drawn for DNA extraction and determination of metabolic parameters. Lipid levels were measured with a Synchron LX-20 automated analyzer (Beckman-Coulter, CA, USA). Approximately 3,200 individuals participated.

MICROS: The MICROS study [32] (<http://www.biomedcentral.com/1471-2350/8/29>) is part of the genomic health care program 'GenNova' and was carried out in three villages of the Val Venosta on the populations of Stelvio, Vallelunga and Martello. This study was an extensive survey carried out in South Tyrol (Italy) in the period 2001 – 2003. Study participants were volunteers from three isolated villages located in the Italian Alps, in a German-speaking region bordering with Austria and Switzerland. Due to geographical, historical and political reasons, the entire region experienced a prolonged period of isolation from surrounding populations. Genotyping was performed on just under 1,400 participants with 1,334 available for analysis after data cleaning. Information on participants’ health status was collected through a standardized questionnaire and clinical examinations.

NSPHS: The Northern Swedish Population Health Study (NSPHS) represents a family-based prospective population study located in the parish of Karesuando, in the subartic region of the County of Norrbotten. This parish has about 1,500 inhabitants, 740 of whom participated in the study. Historic population accounts show that there has been little immigration or other dramatic population change in this area during the last 200 years. The study includes a comprehensive health investigation and collection of data on family structure, lifestyle, diet, medical history and samples for clinical chemistry, RNA and DNA analyses

ORCADES: The Orkney Complex Disease Study (ORCADES) is an ongoing family-based, cross-sectional study in the isolated Scottish archipelago of Orkney. Genetic diversity in this population is decreased compared to Mainland Scotland, consistent with high levels of endogamy historically. Participants included here were aged 18 – 92 years and came from a subgroup of ten islands.

VIS: The Vis study [33] includes unselected Croatians, aged 18 – 93 years, who were recruited during 2003 and 2004 from the villages of Vis and Komiza on the Dalmatian island of Vis. Biochemical and physiological measurements were done, detailed genealogies reconstructed, questionnaire of lifestyle and environmental exposures collected, and blood samples and lymphocytes extracted and stored for further analyses. The 800 participants included in this study were these who had both GWA and phenotypic data available.

**KORA F3 and F4:** The KORA cohorts [34–36] are several cohorts representative of the general population in Augsburg, Southern Germany and two surrounding counties that were initiated as part of the WHO MONICA Study. Ten years age-sex strata have been sampled from the 25 to 74 year old population with a stratum size of 640 subjects. In the KORA S3 study 4,856 subjects (response rate 75%), and in KORA S4 in total 4,261 subjects have been examined (response rate 67%). 3,006 individuals participated in a follow-up examination of S3 in 2004/05 which is called KORA F3. Follow-up for the S4 survey was performed in 3,080 individuals in 2004/2005 (KORA F4). All study participants underwent a standardized face-to-face interview by certified medical staff and a standardized medical examination including blood draw and anthropometric measurements. From KORA F3, 1644 subjects were randomly chosen for analysis and 1,814 subjects for KORA F4. Total cholesterol was determined by cholesterol-esterase method (CHOL Flex, Dade-Behring, Germany), triglycerides and HDL-cholesterol using the TGL Flex and AHDL Flex method (Dade-Behring), respectively, and LDL-cholesterol was measured by a direct method (ALDL, Dade-Behring).

**NFBC1966:** Mothers expected to give birth in the two northern provinces of Oulu and Lapland in 1966 were enrolled in Nothern Finnish Birth Cohort of 1966 [37] (*N* = 12,058 live births). At the 31-year clinical examination, participants provided fasting blood samples for evaluation of the metabolic measures that are the focus of the current study (*N* = 5,654), and DNA was also extracted from the blood samples provided at this time. All DNA samples for the Illumina Infinium 370cnvDuo array were prepared for genotyping by the Broad Institute Biological Sample Repository (BSP) as a part of STAMPEED consortium funded by the US National Heart, Lung, and Blood Institute.

**HBCS:** Helsinki Birth Cohort Study [38, 39] (HBCS) includes 8,760 subjects born in Helsinki between 1934 and 1944. Between 2000 and 2002, a representative subset of 928 males and 1,075 females participated in a clinical study focusing upon cardiovascular and metabolic outcomes and cognitive function.

**YFS:** Cardiovascular Risk of Young Finns Study [40] (YFS) is a prospective cohort study conducted at five university departments of medical schools in Finland (i.e., Turku, Helsinki, Kuopio, Tampere, and Oulu), with the aim of studying the levels of cardiovascular risk factors in children and adolescents in different parts of the country. The first cross-sectional survey was conducted in 1980. Total sample size was 4,320 boys and girls in 6 age cohorts (aged 3, 6, 9, 12, 15 and 18). These subjects were randomly chosen from the national register. A total of 3,596 subjects (83.2% of those invited) participated the study in 1980. After that, several follow-up studies of this cohort have been conducted. The latest follow-up was performed in 2007, when the study subjects had reached the age of 30 to 45 years. In the latest follow-up in 2007 a total of 2,204 subjects were examined.

**FINRISK:** For each FINRISK (1992, 1997, 2002) study [41], a representative random sample is selected from the 25 – 74 year old inhabitants in different regions in Finland. The survey includes a mailed questionnaire and a clinical examination where a blood sample is drawn. A total of 23,036 individuals participated in the cohorts, and gave written informed consent. The genome wide genotype data was available for 2,066 subjects selected as controls for CAD-cases for other study.

**Twin sample.** The Danish, Dutch, Finnish, Italian, Norwegian and Swedish national twin cohorts, together with St. Thomas' twin cohort in Great Britain and an Australian twin cohort, form the basis of the GenomEUtwin project [42], a collection of over 300,000 twins. From these cohorts, we selected female monozygotic pairs aged 20 – 80 years with blood lipid measurements available on both members not overlapping other cohorts. The sample consisted of pairs from, Denmark (173 pairs), Finland (152 pairs), The Netherlands (331 pairs), Sweden (301 pairs) and the UK (462 monozygotic (MZ) pairs and 3,000 dizygotic (DZ) pairs). DNA samples from one member of each monozygotic twin pair were used for genotyping (twin cohort details; S.R. *et al*., unpublished data).

**KORCULA:** The KORCULA study sampled Croatians from the Adriatic island of Korcula, between the ages of 18 and 88. The fieldwork was performed in 2007 in the eastern part of the island, targeting healthy volunteers from the town of Korčula and the villages of Lumbarda, Žrnovo and Račišće. Mortara ELI 350 was used in ECG recording.

**NTR:** The NTR samples include individuals from Dutch twin families from the Netherlands Twin Register (NTR) who participated in the NTR Biobank study. We selected 1,857 unrelated controls at low liability for MDD for genome-wide genotyping with support of the US Foundation for the National Institutes of Health Genetic Association Information Network (FNIH/GAIN). Baseline characteristics of the participants have been previously described [43]. The average age of participants was 43.6 years (65% women) and lipids were measured from fasting blood samples. Participants on lipid lowering medication were excluded. Individual genotyping was conducted by Perlegen Sciences (mountain View, CA, USA) using a set of four proprietary, high-density oligonucleotide arrays. Quality control and data cleaning has been described previously [29].

**NTR2:** GWA data and fasting blood lipid levels were obtained for 1,069 unrelated individuals who participated in the NTR Biobank study [44]. Participants on lipid lowering medication were excluded. For genome-wide SNP-genotyping 200 ng of DNA was hybridized to the Illumina^TM^ Human660W-Quad DNA Analysis BeadChips, containing 660K markers per sample. Experimental procedure was performed according to the Infinium® HD Assay Super manual from Illumina (Illumina, San Diego, USA). Allele calls were determined using Illumina BeadStudio (Illumina, San Diego, USA). Complete genotyping procedure was performed in the Genomics platform (certified service provider (CSPro(R)) for Illumina Inc.) at the LIFE & BRAIN Center Bonn. Quality control excluded SNPs based on MAF < 0.01, missing genotype rate > 0.05 or a *P*-value < 1 x 10^-5^ in a test of Hardy-Weinberg equilibrium. After quality control, 500K SNPs were left (78 %). Samples were excluded if they showed evidence for contamination by excessive allele sharing with multiple samples and excessive levels of heterozygosity (*F* < -0.10). Samples were also excluded if they had a higher than 90% genotype missing rate. Subsequently, genotypes of ~ 3.8 million SNPS were imputed with the IMPUTE program, using the HapMap CEU data (release 22, NCBI build 36), available from the IMPUTE website, as reference. Imputed SNPs were excluded if they had a minor allele frequency < 0.01 or a properinfo < 0.40 and HWE with *P* < 0.00001.

**Rotterdam study:** The Rotterdam Study [45, 46] (I and II) is a prospective cohort study that started in 1990 in Ommoord, a suburb of Rotterdam, among 10,994 men and women aged 55 and over. The main objective of the Rotterdam Study is to investigate the prevalence and incidence and risk factors for cardiovascular, neurological, locomotor and ophthalmologic diseases in the elderly. Baseline measurements were obtained between 1990 and 1993. All participants were subsequently examined in follow-up examination rounds every 2 – 3 years. For this study, we used 6,000 participants for whom GWA data were available; nonfasting total cholesterol and HDL was available for most study participants; fasting TC, HDL and TG were available for 2,300 participants of Rotterdam Study 3 (1997 – 1999). LDL was estimated using the Friedewald formula, eLDL = TC – HDL – 0.456 TG (mmol/l), using nonfasting samples only. Nonfasting TC and HDL and fasting TG and estimated LDL were used in this study. In 2006 a further extension of the cohort was initiated in which 3,932 subjects were included, aged 45 – 54 years, out of 6,057 invited, living in the Ommoord district [47] (RSIII).

**LIFELINES:** The LifeLines Cohort Study [48] is a multi-disciplinary prospective population-based cohort study examining in a unique three-generation design the health and health-related behaviours of 165,000 persons living in the North East region of The Netherlands. It employs a broad range of investigative procedures in assessing the biomedical, socio-demographic, behavioural, physical and psychological factors which contribute to the health and disease of the general population, with a special focus on multimorbidity. In addition, the LifeLines project comprises a number of cross-sectional sub-studies which investigate specific age-related conditions. These include investigations into metabolic and hormonal diseases, including obesity, cardiovascular and renal diseases, pulmonary diseases and allergy, cognitive function and depression, and musculoskeletal conditions. All survey participants are between 18 and 90 years old at the time of enrollment. Recruitment has been going on since the end of 2006, and until March 2011 over 40,000 participants have been included. More information can be found on [www.lifelines.net](http://www.lifelines.net/).

**PREVEND:** The Prevention of REnal and Vascular ENd stage Disease (PREVEND) study [49] is an ongoing prospective study investigating the natural course of increased levels of urinary albumin excretion and its relation to renal and cardiovascular disease. Inhabitants 28 to 75 years of age (*N* = 85,421) in the city of Groningen, The Netherlands, were asked to complete a short questionnaire, 47 % responded, and individuals were then selected with a urinary albumin concentration of at least 10 mg/l (*N* = 7,768) and a randomly selected control group with a urinary albumin concentration less than 10 mg/l (*N* = 3,395). Details of the protocol have been described elsewhere ([www.prevend.org](http://www.prevend.org/)).

**EGCUT:** The Estonian cohort is from the population-based biobank of the Estonian Genome Center of University of Tartu [50, 51]. The whole project is conducted according to the Estonian Gene Research Act and all participants have signed the broad informed consent^1^ ([www.geenivaramu.ee](http://www.geenivaramu.ee/)). The current cohort size is over 50,000, from 18 years of age and up, which reflects closely the age distribution in the adult Estonian population. Subjects are recruited by the general practitioners (GP) and physicians in the hospitals were randomly selected from individuals visiting GP offices or hospitals. Each participant filled out a Computer Assisted Personal interview during 1 – 2 hours at a doctor’s office, including personal data (place of birth, place(s) of living, nationality etc.), genealogical data (family history, three generations), educational and occupational history and lifestyle data (physical activity, dietary habits, smoking, alcohol consumption, women´s health, quality of life).

**Genmets:** The national Health 2000 survey (more information at http://www.terveys2000.fi/indexe.html) was carried out in Finland from fall 2000 to spring 2001. Genmets subset was obtained by collecting all those who fulfilled the IDF definition of metabolic syndrome criteria, and selecting a matched control for each (previously described by Pajunen *et al* [52])

**SORBS:** All subjects are part of a sample from an extensively phenotyped self-contained population from Eastern Germany, the Sorbs [53, 54]. The Sorbs are of Slavonic origin, and lived in ethnic isolation among the Germanic majority during the past 1100 years. Today, the Sorbian-speaking, Catholic minority comprises approximately 15,000 full-blooded Sorbs resident in about 10 villages in rural Upper Lusatia (Oberlausitz), Eastern Saxony.

**CoLaus:** The Cohorte Lausannoise (CoLaus) Study [55] participants were randomly selected from a list of 56,694 individuals aged 35 to 75 years who were permanent residents of the City of Lausanne, Switzerland. Only individuals with four grandparents of European origin were included in the study. The CoLaus study was sponsored in part by GlaxoSmithKline, and all participants were duly informed about this sponsorship. A total of 4,791 participants with total cholesterol and waist-hip ratio measurements were included in this analysis.

**EPIC:** The European Prospective Investigation of Cancer (EPIC)-Norfolk is a population-based cohort study of 25,663 residents of Norfolk, UK, an ethnically homogenous European origin population aged between 39 and 79 years and recruited through general practice age-sex registers [56]. Two subsets of EPIC-Norfolk participants were genotyped with the Infinium HumanHap300 SNP chip (Illumina, San Diego, CA, USA), containing 317,503 tagging SNPs derived from phase I of the International HapMap project. A sample of 2,566 individuals was selected from the total cohort using a random selection algorithm, and a further sample of 1,685 individuals with obesity (BMI ≥ 30 kg/m^2^) of whom 1,284 were non-overlapping and were used as a second study set [57]. After quality control exclusions, a total of 2,005 participants from the cohort sample who were not on lipid lowering therapy and who had measurements for total cholesterol and waist-hip ratio were included in this analysis. For the obese case sample, a total of 1,017 participants (not on lipid lowering therapy and with total cholesterol and waist-hip ratio measurements) were included. Serum total cholesterol was measured in fresh samples with the RA-1000 analyser (Bayer Diagnostics, Basingstoke, UK). The Norwich local research ethics committee granted ethical approval for the study. All participants gave written informed consent.

**Adipose Tissue eQTL dataset:** The fat biopsies were available from individuals who are study subjects of the EUFAM study with dyslipidemic Finnish families [58] . The 54 subcutaneous fat biopsies available were collected from individuals having either very high or low plasma HDL-cholesterol (>90th and <10th age and sex specific population percentiles, respectively) so that the sex distribution in both low and high HDL groups were balanced. No relatives were included within each group.

**A full list of acknowledgments**

**ATFS:** Part of the genotyping was carried out at the Center for Inherited Disease Research, Baltimore (CIDR) through an access award to the late Dr. Richard Todd. Genotype QC and imputation was conducted by the QIMR GWAS Group including Scott Gordon, Sarah Medland, Dale Nyholt and Naomi Wray.

**EUROSPAN:**

MICROS: For the MICROS study in South Tyrol, we thank the primary care practitioners R. Stocker, S. Waldner, T. Pizzecco, J. Plangger, U. Marcadent and the personnel of the Hospital of Silandro (Department of Laboratory Medicine) for their participation and collaboration in the research project.

ERF: We are grateful to all study participants and their relatives, general practitioners and neurologists for their contributions and to P. Veraart for the help in genealogy, J. Vergeer for the supervision of the laboratory work and P. Snijders for his help in data collection.

NSPHS: We are grateful for the contribution of district nurse Svea Hennix for data collection and Inger Jonasson for logistics and coordination of the health survey. Finally, we would like to thank all the participants from the community for their interest and willingness to contribute to this study.

**KORA F3 and F4**: We gratefully acknowledge the contribution of P. Lichtner, G. Eckstein and T. Strom and all other members of the Helmholtz Zentrum München genotyping staff in generating and analyzing the SNP dataset. We thank all members of field staffs who were involved in the planning and conduct of the MONICA/KORA Augsburg studies. Finally we express our appreciation to all study participants.

**NFBC1966:** The DNA extractions, sample quality controls, biobank up-keeping and aliquotting of NFBC1966 was performed in the National Public Health Institute, Biomedicum Helsinki, Finland.

**TWINSUK:** We thank the staff from the TwinsUK, the DNA Collections and Genotyping Facilities at the Wellcome Trust Sanger Institute for sample preparation; Quality Control of the Twins UK cohort for genotyping (in particular Amy Chaney, Radhi Ravindrarajah, Douglas Simpkin, Cliff Hinds, and Thomas Dibling); Paul Martin and Simon Potter of the DNA and Genotyping Informatics teams for data handling; Le Centre National de Génotypage, France, led by Mark Lathrop, for genotyping; Duke University, North Carolina, USA, led by David Goldstein, for genotyping; and the Finnish Institute of Molecular Medicine, Finnish Genome Center, University of Helsinki.

**Rotterdam Study:** We thank Pascal Arp, Mila Jhamai, Marijn Verkerk, Lizbeth Herrera and Marjolein Peters for their help in creating the GWAS database, and Karol Estrada for the support in creation and analysis of imputed data. The authors are grateful to the study participants, the staff from the Rotterdam Study and the participating general practitioners and pharmacists.

**LIFELINES**: We thank Behrooz Alizadeh, Annemieke Boesjes, Marcel Bruinenberg, Noortje Festen, Ilja Nolte, Lude Franke, Mitra Valimohammadi for their help in creating the GWAS database, and Rob Bieringa, Joost Keers, René Oostergo, Rosalie Visser, Judith Vonk for their work related to data-collection and validation. The authors are grateful to the study participants, the staff from the LifeLines Cohort Study and Medical Biobank Northern Netherlands, and the participating general practitioners and pharmacists. LifeLines Scientific Protocol Preparation: Rudolf de Boer, Hans Hillege, Melanie van der Klauw, Hans Ormel, Dirkje Postma, Judith Rosmalen, Joris Slaets, Ronald Stolk; LifeLines GWAS Working Group: Behrooz Alizadeh, Marike Boezen, Marcel Bruinenberg, Noortje Festen, Lude Franke, Dirkje Postma, Harold Snieder.

**EGCUT:** EGCUT authors want to thank Viljo Soo for his contribution in genotyping.

**SORBS:** The Sorbs authors would like to thank Knut Krohn from the Microarray Core Facility of the Interdisciplinary Centre for Clinical Research (IZKF), University of Leipzig, Germany, for his excellent genotyping support and Nigel William Rayner from the Wellcome Trust Centre for Human Genetics, University of Oxford, UK, as well as John Broxholm from the Bioinformatics Core Unit of the Wellcome Trust Centre for Human Genetics for their excellent bioinformatics support.

**CoLaus:** The authors thank Peter Vollenweider, Vincent Mooser and Gereard Waeber, Co-PIs of the CoLaus study. Special thanks to Murielle Bochud, Yolande Barreau, Mathieu Firmann, Vladimir Mayor, Anne-Lise Bastian, Binasa Ramic, Martine Moranville, Martine Baumer, Marcy Sagette, Jeanne Ecoffey and Sylvie Mermoud for data collection.

**Adipose Tissue eQTL dataset:** The authors thank Sanni Söderlund, Jukka Westerbacka, Helinä Perttunen-Nio and Lea Puhakka.

**Supplementary References**

27. Middleberg R, Martin N and Whitfield J (2007) A longitudinal genetic study of plasma lipids in adolescent twins. Twin Res Hum Genet 10: 127-135.

28. Heath A, Whitfield J, Martin N, Pergadia M, Goate A, et al. (2011) A quantitative-trait genome-wide association study of alcoholism risk in the commnity: findings and implications. Biol Psychiatry

29. Sullivan P, de Geus E, Willemsen G, James M, Smit J, et al. (2009) Genome-wide association for major depressive disorder: a possible role for the presynaptic protein piccolo. Mol Psychiatry 14: 359-375.

30. Painter J, Anderson C, Nyholt D, Macgregor S, Lin J, et al. (2011) Genome-wide association study identifies a locus at 7p15.2 associated with endometriosis. Nat Genet 43: 51-54.

31. Pardo L, MacKay I, Oostra B, van Duijn C and Aulchenko Y (2005) The effect of genetic drift in a young genetically isolated population. Ann Hum Genet 69: 288-295.

32. Pattaro C, Marroni F, Riegler A, Mascalzoni D, Pichler I, et al. (2007) The genetic study of three population microisolates in South Tyrol (MICROS): study design and epidemiological perspectives. BMC Med Genet 8: 29.

33. Rudan I, Campbell H and Rudan P (1999) Genetic epidemiological studies of eastern Adriatic Island isolates, Croatia: objective and strategies. Coll Antropol 23: 531-546.

34. Wichmann H, Gieger C, Illig T and MONICA/KORA Study Group (2005) KORA-gen--resource for population genetics, controls and a broad spectrum of disease phenotypes. Gesundheitswesen 67 Suppl 1: S26-30.

35. Heid I, Boes E, Müller A, Kollerits B, Lamina C, et al. (2008) Genome-wide association analysis of high-density lipoprotein cholesterol in the population-based KORA Study sheds new light on intergenic regions. Circulation: Cardiovascular Genetics 1: 10-20.

36. Kollerits B, Coassin S, Beckmann N, Teumer A, Kiechl S, et al. (2009) Genetic evidence for a role of adiponutrin in the metabolism of apolipoprotein B-containing lipoproteins. Hum Mol Genet 18: 4669-4676.

37. Sabatti C, Service S, Hartikainen A, Pouta A, Ripatti S, et al. (2009) Genome-wide association analysis of metabolic traits in a birth cohort from a founder population. Nat Genet 41: 35-46.

38. Ylihärsilä H, Kajantie E, Osmond C, Forsén T, Barker D, et al. (2008) Body mass index during childhood and adult body composition in men and women aged 56-70 y. Am J Clin Nutr 87: 1769-1775.

39. Eriksson J, Forsén T, Tuomilehto J, Osmond C and Barker D (2001) Early growth and coronary heart disease in later life: longitudinal study. BMJ 322: 949-953.

40. Raitakari O, Juonala M, Rönnemaa T, Keltikangas-Järvinen L, Räsänen L, et al. (2008) Cohort profile: the cardiovascular risk in Young Finns Study. Int J Epidemiol 37: 1220-1226.

41. Vartiainen E, Laatikainen T, Peltonen M, Juolevi A, Männistö S, et al. (2010) Thirty-five-year trends in cardiovascular risk factors in Finland. Int J Epidemiol 39: 504-518.

42. Peltonen L and GenomEUtwin (2003) GenomEUtwin: a strategy to identify genetic influences on health and disease. Twin Res 6: 354-360.

43. Boomsma D, Willemsen G, Sullivan P, Heutink P, Meijer P, et al. (2008) Genome-wide association of major depression: description of samples for the GAIN Major Depressive Disorder Study: NTR and NESDA biobank projects. Eur J Hum Genet 16: 335-342.

44. Willemsen G, de Geus E, Bartels M, van Beijsterveldt C, Brooks A, et al. (2010) The Netherlands Twin Register biobank: a resource for genetic epidemiological studies. Twin Res Hum Genet 13: 231-245.

45. Hofman A, Breteler M, van Duijn C, Krestin G, Pols H, et al. (2007) The Rotterdam Study: objectives and design update. Eur J Epidemiol 22: 819-829.

46. Hofman A, Grobbee D, de Jong P and van den Ouweland F (1991) Determinants of disease and disability in the elderly: the Rotterdam Elderly Study. Eur J Epidemiol 7: 403-422.

47. Hofman A, Breteler M, van Duijn C, Janssen H, Krestin G, et al. (2009) The Rotterdam Study: 2010 objectives and design update. Eur J Epidemiol 24: 553-572.

48. Stolk R, Rosmalen J, Postma D, de Boer R, Navis G, et al. (2008) Universal risk factors for multifactorial diseases: LifeLines: a three-generation population-based study. Eur J Epidemiol 23: 67-74.

49. Hillege H, Fidler V, Diercks G, van Gilst W, de Zeeuw D, et al. (2002) Prevention of Renal and Vascular End Stage Disease (PREVEND) Study Group. Urinary albumin excretion predicts cardiovascular and noncardiovascular mortality in general population. Circulation 206: 1777-1782.

50. Metspalu A (2004) The Estonian Genome Project. Drug Rev Res 62: 97-101.

51. Nelis M, Esko T, Mägi R, Zimprich F, Zimprich A, et al. (2009) Genetic structure of Europeans: a view from the North-East. PLoS One 4: e5472.

52. Pajunen P, Rissanen H, Härkänen T, Jula A, Reunanen A, et al. (2010) The metabolic syndrome as a predictor of incident diabetes and cardiovascular events in the Health 2000 Study. Diabetes Metab 36: 395-401.

53. Tönjes A, Koriath M, Schleinitz D, Dietrich K, Böttcher Y, et al. (2009) Genetic variation in GPR133 is associated with height: genome wide association study in the self-contained population of Sorbs. Hum Mol Genet 18: 4662-4668.

54. Tönjes A, Zeggini E, Kovacs P, Böttcher Y, Schleinitz D, et al. (2010) Association of FTO variants with BMI and fat mass in the self-contained population of Sorbs in Germany. Eur J Hum Genet 18: 104-110.

55. Firmann M, Mayor V, Vidal P, Bochud M, Pécoud A, et al. (2008) The CoLaus study: a population-based study to investigate the epidemiology and genetic determinants of cardiovascular risk factors and metabolic syndrome. BMC Cardiovasc Disord Mar 17;8: 6.

56. Day N, Oakes S, Luben R, Khaw K, Bingham S, et al. (1999) EPIC-Norfolk: study design and characteristics of the cohort. European Prospective Investigation of Cancer. Br J Cancer 80 Suppl 1: 95-103.

57. Sandhu M, Waterworth D, Debenham S, Wheeler E, Papadakis K, et al. (2008) LDL-cholesterol concentrations: a genome-wide association study. Lancet 371: 483-491.

58. Pajukanta P, Porkka K, Antikainen M, Taskinen M, Perola M, et al. (1997) No evidence of linkage between familial combined hyperlipidemia and genes encoding lipolytic enzymes in Finnish families. Arterioscler Thromb Vasc Biol 17: 841-850.
